# Transcriptome Sequence of the Bloodstream Form of *Trypanoplasma borreli*, a Hematozoic Parasite of Fish Transmitted by Leeches

**DOI:** 10.1128/genomeA.01712-16

**Published:** 2017-03-02

**Authors:** Mark Carrington, Eva Dóró, Maria Forlenza, Geert F. Wiegertjes, Steven Kelly

**Affiliations:** a Department of Biochemistry, University of Cambridge, Cambridge, United Kingdom; b Cell Biology and Immunology Group, Department of Animal Sciences, Wageningen University, Wageningen, The Netherlands; c Department of Plant Sciences, University of Oxford, Oxford, United Kingdom

## Abstract

Here, we report a transcriptome sequence of *Trypanoplasma borreli* isolated from its natural host, the common carp, *Cyprinus carpio*. The transcriptome allows an analysis of abundant cell surface proteins and acts as a comparator for understanding the evolution and pathogenicity of other *Kinetoplastida*, including several that infect humans.

## GENOME ANNOUNCEMENT

The *Kinetoplastida* comprise a class of protozoa that separated early in the evolutionary diversification of eukaryotes (1). Within the *Kinetoplastida*, the *Trypanosomatidae* comprise a monophyletic group of parasites and pathogens that have evolved to colonize a diverse range of eukaryotic hosts (2). Most *Trypanosomatidae* spp. are transmitted between hosts by biting insects, and several species cause economic and health losses in developing countries (3). The *Bodonida* comprise a second group within the *Kinetoplastida* and are more diverse with free-living, commensal, and pathogenic species (4, 5). Here, we report a transcriptome assembly from a pathogenic *Bodonida* species, *Trypanoplasma borreli* (taxon ID 5710), isolated from experimentally infected carp. *T. borreli* infection can cause severe anemia and splenomegaly in both wild and farmed fish (6).

The isolate used here originated from a common carp, *Cyprinus carpio*, in a hatchery near Celle in Germany and was cloned in 1984 at the Hannover Medical School by the Fish Disease Research Unit at Tierärztliche Hochschule in Hannover, Germany (6, 7), and has been maintained in laboratories through serial infections of carp and stored as stabilates in liquid nitrogen. *T. borreli* was grown in carp to a parasitemia of ~1 × 10^8^/ml and isolated from heparinized blood (8). Total RNA was prepared using the QIAgen RNeasy protocol. A library was made from polyA-selected RNA using random hexamer priming for reverse transcriptase. Sequencing was performed on an Illumina HiSeq using a single library of 90-nucleotide paired-end reads with a 170-bp inset size. Quality-filtered reads were assembled into contigs using the string graph assembler (SGA) (9). Contigs were then subject to scaffolding using SSPACE (10) and the full set of reads using the settings -k, 10; -a, 0.7; -n, 50; and -o, 20. Scaffolds were subject to gap filling using the SGA gap-filling function. Gene models were predicted using GeneMark-ST (11) and clustered into orthogroups using OrthoFinder (12). The assembled transcriptome contained 15,713 contigs encoding 13,640 putative proteins greater than 100 amino acids in length.

### Accession number(s).

This transcriptome shotgun assembly project has been deposited at GenBank under the accession number GFCF00000000. The version described in this paper is the first version, GFCF01000000. The raw reads are available from NCBI SRA under the accession number SRX2395293.
